# The association between HIV (treatment), pregnancy serum lipid concentrations and pregnancy outcomes: a systematic review

**DOI:** 10.1186/s12879-017-2581-8

**Published:** 2017-07-11

**Authors:** Marissa J. Harmsen, Joyce L. Browne, Francois Venter, Kerstin Klipstein-Grobusch, Marcus J. Rijken

**Affiliations:** 1 Julius Global Health, Julius Center for Health Sciences and Primary Care, University Medical Center Utrecht, Utrecht, the Netherlands; 2 Wits Reproductive Health and HIV Institute, Faculty of Health Sciences, University of the Witwatersrand, Johannesburg, South Africa; 3 Division of Epidemiology & Biostatistics, School of Public Health, Faculty of Health Sciences, University of the Witwatersrand, Johannesburg, South Africa; 4 Department of Obstetrics and Gynaecology, University Medical Center Utrecht, Utrecht, The Netherlands

**Keywords:** HIV, ART, Lipids, Pregnancy

## Abstract

**Background:**

Observed adverse effects of antiretroviral therapy (ART) on the lipid profile could be of significance in pregnancy. This systematic review aims to summarize studies that investigated the association between HIV, ART and serum lipids during pregnancy and adverse pregnancy outcomes.

**Methods:**

A systematic search was conducted in five electronic databases to obtain articles that measured serum lipid concentrations or the incidence of dyslipidaemia in HIV-infected pregnant women. Included articles were assessed for quality according to the Cochrane Risk of Bias Tool. The extracted data was analysed through descriptive analysis.

**Results:**

Of the 1264 articles screened, 17 articles were included in this review; eleven reported the incidence of dyslipidaemia, and twelve on maternal serum lipid concentrations under the influence of HIV-infection and ART. No articles reported pregnancy outcomes in relation to serum lipids. Articles were of acceptable quality, but heterogenic in methods and study design. Lipid levels in HIV-infected women increased 1.5–3 fold over the trimesters of pregnancy, and remained within the physiological reference range. The percentage of women with dyslipidaemia was variable between the studies [0–88.9%] and highest in the groups on first generation protease inhibitors and for women on ART at conception.

**Conclusion:**

This systematic review observed physiologic concentrations of serum lipids for HIV-infected women receiving ART during pregnancy. Serum lipids were increased in users of first generation protease inhibitors and for those on treatment at conception. There was no information available about pregnancy outcomes. Future studies are needed which include HIV-uninfected control groups, control for potential confounders, and overcome limitations associated with included studies.

**Electronic supplementary material:**

The online version of this article (doi:10.1186/s12879-017-2581-8) contains supplementary material, which is available to authorized users.

## Background

Globally over 16 million women of reproductive age live with human immunodeficiency virus (HIV), of whom most in sub-Saharan Africa (SSA) [1]. Among young women in SSA, HIV prevalence is almost three times higher compared to their male counterparts [1]. Optimizing preventive HIV care for these women is essential, as many of them may become pregnant in the near future. Mother-to-child transmission (MTCT) can be reduced to <5% in breastfeeding, and <2% in non-breastfeeding HIV-infected pregnant women with controlled plasma HIV RNA levels [2]. Through the more widespread availability of antiretroviral therapy (ART), 1.5 million pregnant women - 73% of all pregnant women living with HIV globally - received ART in 2014 [3].

While women represent half of the HIV-infected population worldwide, uncertainty remains about the effects of ART in women as they represented a mere 20% of the subjects in ART clinical trials [4]. Systematic reviews of ART trials observed similar efficacy of ART in males and females, but reduced tolerability and more side effects in women [4–7]. Other studies showed increased levels of total cholesterol (TC), low-density lipoprotein cholesterol (LDL-C) and triglycerides (TG) in women under ART, diminishing the protective effect of the female sex against atherosclerosis [8]. Physiological and metabolic changes associated with pregnancy could further influence the pharmacokinetics of ART [7, 9].

The impact of antiretroviral therapy on lipid profiles, especially first generation protease inhibitors, has been linked to increased rates of cardiovascular complications. This association was not seen for the second generation protease inhibitors [10]. Pregnant women on protease inhibitor (PI)-based ART were reported to have higher TG levels than those on non-PI based ART [11]. In the general population, first generation PIs, such as indinavir and lopinavir, and non-nucleoside reverse transcriptase inhibitors (NNRTI), such as efavirenz, resulted in higher increases in TC, LDL-C and TG than second generation PIs such as atazanavir and darunavir [10, 12]. The adverse effect of NNRTI use on the lipid profile is counterbalanced by an increase in HDL-C. Particularly nevirapine is associated with a decline in TG levels and a pronounced increase in HDL-C [13, 14].

The potential adverse effect of ART on lipid profiles may have consequences in pregnancy. Elevated levels of TC, non-HDL-C, and TG have been associated with pre-eclampsia in non-HIV-infected women [15, 16]. A large European cohort study observed atherogenic lipid profiles (elevated TC and TG) in the first trimester of pregnancy to be associated with an increased risk of adverse pregnancy outcomes such as gestational hypertension, pre-eclampsia and preterm birth [17, 18]. This suggests that lipids could be a target to prevent adverse maternal and perinatal outcomes [19, 20], and additional insight in the relationship between serum lipids and pregnancy (outcomes) in relation to HIV-infection and its treatment could support the management of pregnant HIV-infected women. Therefore, the aim of this systematic review is to summarize the studies that investigated the association between HIV, ART and serum lipids during pregnancy and adverse pregnancy outcomes.

## Methods

### Search strategy and eligibility criteria

This systematic review was written following PRISMA guidelines [21]. The review protocol was registered with the registry for systematic reviews PROSPERO (ID: CRD42015024729) on 21 July 2015. Studies were eligible when maternal serum lipids in HIV-infected women were measured. Excluded were animal studies, biomolecular studies, publications not written in English, French, German, Spanish, or Dutch, case reports, reports of proceedings, conference abstracts and secondary analyses.

A systematic literature search was conducted in the following electronic bibliographic databases: PubMed/MEDLINE, The Cochrane Library (Cochrane Database of Systematic Reviews), EMBASE, Global Health Library, and Popline, up to 21 July 2015. A combined text and MeSH search strategy of terms related to HIV/AIDS and ART, serum lipids and pregnancy was used (see Additional file 1 for the full search strategy). There were no restrictions for dates, study design, type of facility or geographical location in the initial search. All reference lists of eligible studies were searched for additional studies. The screening of the articles on title and abstract was performed independently by two reviewers (MJH and MJR). Any discrepancies between the two reviewers in this process were discussed, and full text accessed when further clarification was required. If discrepancies continued to exist, a third independent reviewer (KKG) was consulted and the article discussed among the researchers until consensus was reached. In case of duplicate publications from the same database, the most completely reporting article was included. The corresponding author was approached once if full text articles were unavailable or data was incomplete.

Data was collected using a standardized data extraction form. This process was performed by a single reviewer (MJH) who was not blinded for journal or author details. A second and third reviewer (MJR and JLB) were approached when more clarity was needed. Data was extracted on study design, −setting, country, population (age and parity), number of patients included, number of controls, gestational age, BMI, HIV severity (CD4 count), type of ART, type of serum lipids measured, study outcome, pregnancy outcome, serum lipid concentrations and rates of dyslipidaemia in HIV-infected ART recipients and control groups.

### Quality assessment

Quality of the included articles was scored according to the Cochrane Risk of Bias Tool [22] (see Additional file 2 for the complete quality assessment). Bias was assessed on the study level, including the selection of the study population, completeness of data, origin of the data (measurements by authors or database research), blinding of the researchers/clinicians, definition of outcome, and confounders. Comparability was evaluated regarding the ART regimen, measured serum lipids and outcome measure of dyslipidaemia. Bias risk was assigned as low, unclear, or high risk, and assigned 2, 1, or 0 points accordingly. The overall quality of the articles was based on the total score; <6 points low quality, ≥6 to ≤10 points acceptable quality, >10 points good quality.

### Data synthesis and statistical analysis

Due to the heterogeneity of the data, a meta-analysis could not be performed and the extracted data on the association between HIV, ART, and serum lipids, as TG, TC, HDL-C and LDL-C was summarized in a descriptive analysis. The data was categorized by trimester based on the mean gestational age at blood sampling. First, second and third trimester was defined as ≥1 to ≤13, ≥14 to ≤26, and ≥27 or more weeks of gestation, respectively. All lipid measurements were reported as milligrams per decilitre (mg/dl) and summarized in scatterplots using SPSS (version 23.0, IBM Corp, Armonk, NY) [23]. Due to the heterogenic nature of methods and study design, no funnel plot of studies could be created.

## Results

**Fig. 1:**
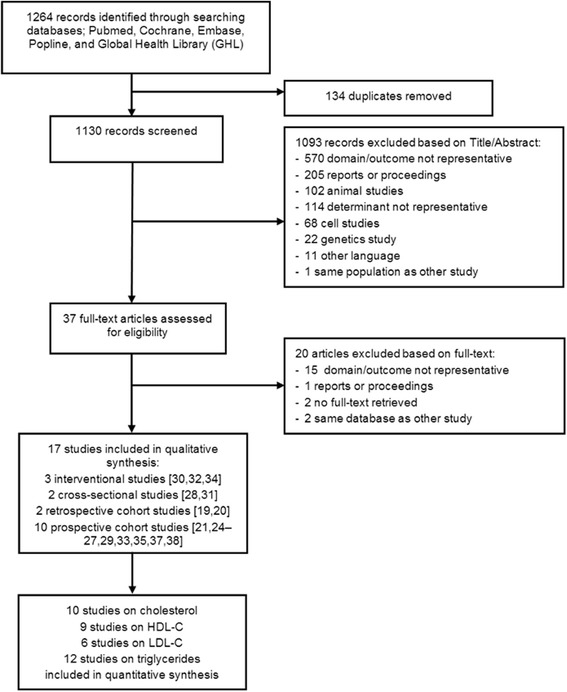
Flow chart

The systematic search identified 1264 publications, of which 37 studies remained after title and abstract screening (Fig. 1). No additional articles were identified through reference checking. Four studies using the same patient database were identified, [24–27] of which the two most recent studies were used [26, 27]. After analysing the full-texts, 17 articles were included in this review. No studies evaluated HIV-infection, ART and dyslipidaemia in relation to pregnancy outcome.

Study characteristics are presented in Table 1. The studies were published between 2006 and 2015 and reported about 20 to 428 HIV-infected pregnant women (total HIV-infected *n* = 2324, total HIV-uninfected *n* = 56). Eleven studies reported about the association of ART in pregnancy and dyslipidaemia. Twelve studies presented data on various serum lipid concentrations in pregnancy under the influence of HIV-infection and ART. The studies were conducted in South America (41%, *n* = 7), the United States (12%, *n* = 2), Nigeria (6%, *n* = 1), Thailand (12%, *n* = 2), and Italy (29%, *n* = 5).

**Table 1:** Characteristics of studies included in the systematic review (*n* = 17)

First author, Year	Country	n exposed	n non-exposed	Exposed/non-exposed	Serum lipid levels in mg/dL: Mean ± SD or Median (IQR)								% dyslipidemia			
					Total cholesterol		HDL-C		LDL-C		Triglycerides					
					cases	controls	cases	controls	cases	controls	cases	controls	All	TC	TG	HDL
First trimester																
Luzi, 2013 [40]	Italy	14	19	HIV+/HIV-	160 (106–215)	154 (134–159)	54 (52–63)^*^	71 (61–82)^*^	85 (51–136)	70 (64–79)	106 (51–212)^*^	54 (47–72)^*^	-	-	-	-
Nasi, 2011 [38]	Italy	68	23	HIV+/HIV-	176 ± 40	-	58 ± 11	-	-	-	103 ± 42	-	-	-	-	-
Second trimester																
Areechokchai, 2009 [41]^*^	Thailand	246	none	-	-	-	-	-	-	-	-	-	1.2	-	-	-
Bonafe, 2013 [44]	Brazil	31	32	LPV/r increased/conventional dose	185	186	56	57	95	90	186	193	-	-	2	-
El Beitune, 2006 [29]	Brazil	25	20	PI-based/only ZDV regimen	-	-	-	-	-	-	177 ± 12^*^	135 ± 11^*^	-	-	-	-
Floridia, 2009 [26]	Italy	86	289	NVP/no NVP	209	206	77^**^	64^**^	110	108	151	169	-	25.6	24.5	6.2
Luzi, 2013 [40]	Italy	14	19	HIV+/HIV-	191 (143–289)^*^	238 (182–278)^*^	67 (59–101)	75 (65–91)	104 (63–150)^*^	128 (82–172)^*^	153 (89–554)	119 (96–187)	-	-	-	-
Machado, 2013 [31]	Brazil	49	none	-	162 (145–177)	-	52 (47–64)	-	-	-	131 (90–180)	-	0	-		
Nasi, 2011 [38]	Italy	68	23	HIV+/HIV-	220 ± 51	-	79 ± 42	-	-	-	183 ± 82	-	-	-	-	-
Omo-Aghoja, 2010 [32]^*^	Nigeria	154	150	pregnant/non-pregnant	-	-	-	-	-	-	-	-	0	-	-	-
Peixoto, 2011 [33]	Brazil	164	70	LPV/r increased/ conventional dose	220 ± 53^*^	205 ± 42^*^	-	-	-	-	235 ± 93	232 ± 106	-	8.6/ 2.5	0/0.6	-
Santini-Oliveira, 2014 [34]^*^	Brazil	27	26	LPV/r increased/ conventional dose	-	-	-	-	-	-	-	-	-	23.1/18.5	11.6/ 11.1	
Third trimester																
Agostini, 2008 [28]	Argentina	29	none	-	-	-	-	-	-	-	-	-	-	62.1	62.1	-
Cade, 2015 [35]	US	16	14	HIV+/HIV-	179 ± 38	205 ± 39	60 ± 12	66 ± 16	87 ± 37	109 ± 23	160 ± 61	150 ± 36	-	-	-	-
Calza, 2012 [42]	Italy	21	20	pregnant/non-pregnant	189 ± 59	171 ± 45	49 ± 21	45 ± 19	112 ± 32	104 ± 41	209 ± 105	226 ± 117	-	29	48	-
Duran, 2006 [37]	Argentina	351	none	-	-	-	-	-	-	-	-	-	-	12.2	7.4	-
El Beitune, 2006 [29]	Brazil	25	20	PI-based/only ZDV regimen	-	-	-	-	-	-	227 ± 17^*^	176 ± 14^*^	-	-	-	-
Floridia, 2014 [27]	Italy	322	106	lopinavir/atazanavir	239 (201–272)^**^	221 (194–250)^**^	65 (56–75)	64 (57–73)	124 (97–154)	115 (90–145)	226 (182–309)^**^	181 (142–236)^**^	-	-	-	-
Livingston 2007 [30]	US	81	77	PI/no PI	230 (197–159)^*^	212 (179–246)^*^	61 (50–69)	63 (54–74)	-	-	224 (187–288)^**^	185 (142–230)^**^	-	-	-	-
Luzi, 2013 [40]	Italy	14	19	HIV+/HIV-	200 (136–258)^**^	302 (272–331)^**^	71 (55–141)	67 (58–90)	111 (41–155)^**^	177 (111–195)^**^	191 (105–370)	178 (132–383)	-	-	-	-
Nasi, 2011 [38]	Italy	68	23	HIV+/HIV-	232 ± 63	-	73 ± 18	-	-	-	231 ± 117	-	-	-	-	-
Ramautarsing 2011 [36]	Thailand	20	none	-	-	-	-	-	-	-	-	-	-	5	5	-

mean ± SD or median (interquartile range IQR)^*^ *P* < 0.05, ^**^ *P* < 0.001, *TC* total cholesterol, *HDL-C* high density lipoprotein cholesterol, *LDL*-*C* low density lipoprotein cholesterol, *TG* triglycerides, *LPV/r* lopinavir/ritonavir, *PI* protease inhibitor, *ZDV* zidovudine, *NVP* neviparine

### Bias assessment

**Fig. 2:**
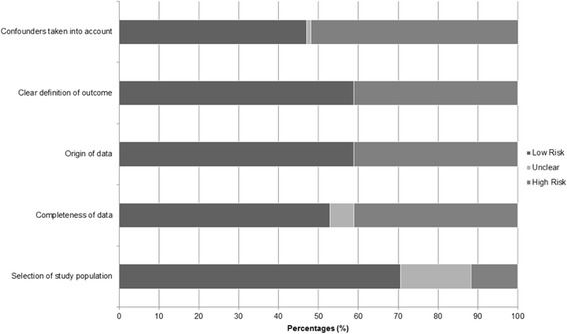
Assessment of risk of bias

The risk assessment of all included studies is summarized in Fig. 2. The individual study risk of bias assessment is available as Supplemental Data (S3 File). The quality of the studies was acceptable. A high risk of bias arose from studies that did not mention [28–34] or control [35–37] for confounders in their analysis (53%, *n* = 9). Other studies did not provide a definition of outcome (41%, *n* = 7), [28, 29, 31–33, 37, 38] used data originating from hospital databases (41%, *n* = 7) or had missing data (41%, *n* = 7). Most studies selected a study population that was representative of the target population (71%, *n* = 12).

### Serum lipid concentrations in pregnancy

**Fig. 3:**
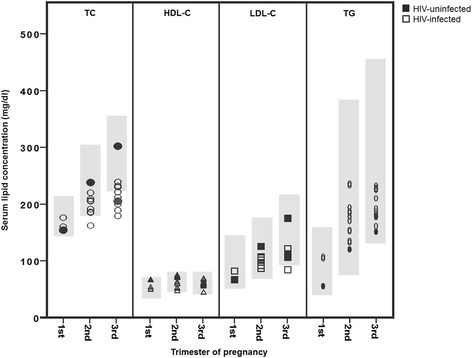
Serum lipid concentrations per trimester of pregnancy. TC total cholesterol, HDL-C high density lipoprotein cholesterol, LDL-C low density lipoprotein cholesterol, TG triglycerides. Serum lipid concentrations from studies in HIV-infected and HIV-uninfected participants represented by open and closed figures respectively. Shaded areas mark reference values for serum lipid concentrations per trimester in a normal (HIV-uninfected) pregnancy [39]

Serum lipid concentrations measured in HIV positive pregnant women are presented in Table 1. In Fig. 3 the serum lipid concentrations per trimester are related to reference values for serum lipid concentrations in pregnancy [39]. Two studies measured serum lipid concentrations in all trimesters [38, 40]. In two studies serum lipids in HIV-infected and -uninfected pregnant women were compared [35, 40]. Cade et al. [35] studied 16 HIV-infected and 14 -uninfected pregnant women who were of similar age, height, weight, and gestational weight gain (GWG) in the third trimester of pregnancy and found serum lipids to be comparable. Luzi et al. [40] included 14 HIV-infected (8 (57%) African) and 19 –uninfected (100% Caucasian) pregnant women of similar age and found that TC and LDL-C were significantly higher in the HIV-uninfected group compared to the HIV-infected group in the second and third trimester. TGs were significantly higher in the HIV-infected group compared to the HIV-uninfected group in the first trimester.

### Dyslipidaemia in relation to ART use in pregnancy

**Fig. 4:**
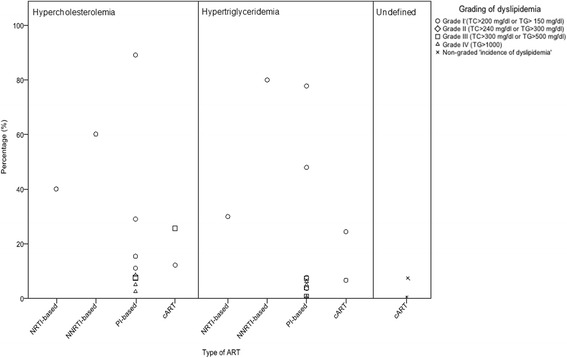
Incidence of dyslipidemia per ART regimen found in individual studies. Incidence of hypercholesterolemia, hypertriglyceridemia, and undefined dyslipidemia per antiretroviral treatment (ART) regimen. NRTI nucleoside reverse transcriptase inhibitor, NNRTI non-nucleoside reverse transcriptase inhibitor, PI protease inhibitor, cART combined antiretroviral treatment. Grading of dyslipidemia according to DAIDS [39], or non-graded incidence of dyslipidemia not specifying the definition of ‘dyslipidemia.’ (crosses) [31, 32, 41]

Table 2 and Fig. 4 provide an overview of the studies that assessed the incidence of dyslipidaemia (total HIV infected women *n* = 1515, total HIV-uninfected women *n* = 0).

**Table 2:** Specifications of ART regimen, the incidence of dyslipidemia and pregnancy outcome in HIV-infected pregnancies (*n* = 17)

First author, Year	n		ART regimen			Grading of Dyslipidemia	% dyslipidemia				Pregnancy outcome			
		ART during ANC %	NRTI %	NNRTI %	PI%		All	TC	TG	HDL-C	PTB n(%)	Stillbirth n(%)	LBW n(%)	PE n(%)
Agostini 2008 [28]	29	100%	100%^c^	34.4%^c^	30.9%^c^	*TC > 200, TG > 150 mg/dl*	-	62.1	62.1	-	-	-	-	2 (6.9)
	10^a^	100%	100%^c^	-	-		-	40.0	30.0	-	-	-	-	-
	10^a^	100%	100%^c^	100%^c^	-		-	80.0	60.0	-	-	-	-	-
	9^a^	100%	100%^c^	-	100%^c^		-	88.9	77.8	-	-	-	-	-
Areechokchai 2009 [41]	246	100%				*UNS*	1.2	-	-	-	25 (10.2)	1 (0.4)	49 (20)	4 (1.6)
	40^a^	16.3% cART	100%^a,b^	>50%^a^	>20%^a^		7.5	-	-	-	(19.4)	-	0	3.2%
	164^a^	66.7% PMTCT	99%^a^	99%^d^	-		0	-	-	-	(6.9)	-	0	-
	42^a^	17.1% ART in labor	95.2%^a^	95.2%^d^	-		0	-	-	-	(19)	-	(19)	-
Bonafe 2013 [44]	32	100%	100%^c^	-	100%^a^	*Grade III*	-	-	6.3	-	(19.3)	-	-	-
	31	100%	100%^c^	-	100%^d^		-	-	0	-	(17.8)	-	-	-
Cade 2015 [35]	16	100%	75%^a^/25%^c^	-	100%^c^	-	-	-	-	-	-	-	-	-
Calza 2012 [42]	21	100%	100%^a^	-	100%^a^	*TC > 200, TG > 250 mg/dl*	-	29	48	-	-	-	-	-
Duran 2006 [37]	271^*^	100%	-	-	-	*Grade I/II*	-	12.2/0	6.7/0.7	-	-	6 (1.7)	-	-
	133^a^	49.1% cART	100%^c^	30.8%^a^	28.6%^a^		-	-	-	-	-	-	-	-
	33^a^	12.2% PMTCT	100%^a^	-	-		-	-	-	-	-	-	-	-
El Beitune 2006 [29]	25	100%	100%^a^	-	100%^a^	-	-	-	-	-	-	-	-	-
	20	100%	100%^a^	-	-		-	-	-	-	-	-	-	-
Floridia 2009 [26]	375	75.4%	UNS	22.9%^a^	39.9%^c^	*TC > 240, TG > 200 mg/dl*	-	25.6	24.5	6.2	-	-	-	-
	86^a^	75.4%	UNS	100%^a^	-		-	-	-	-	-	-	-	-
Floridia 2014 [27]	322	100%	97.8%	-	100%^a^	-	-	-	-	-	69 (21.4)	(<2)	63 (20.8)	-
	106	100%	98.1%	-	100%^e^		-	-	-	-	20 (18.9)	(<2)	24 (23.3)	-
Livingston 2007 [30]	81	100%	84/77%^a^	2.0%^a^	100%^a^	-	-	-	-	-	8 (10)	-	11 (14)	-
	77	96.10%	88/91%^a^	52.0%^a^	-		-	-	-	-	5 (7)	-	9 (12)	-
Luzi, 2013	14	100%	100%^a^	7.1%^c^	92.9%^c^	-	-	-	-	-	-	-	-	-
Machado 2013 [31]	49	69%	-	-	-	*UNS*	0	-	-	-	-	-	-	-
Nasi 2011 [38]	68	100%	100%^a,b^	55.9%^a^	54.4%^a^	-	-	-	-	-	-	-	-	-
Omo-Aghoja 2010 [32]	154	100%	-	-	-	*UNS*	0	-	-	-	-	-	-	-
Peixoto 2011 [33]	164	100%	-	-	100%^a^	*Grade III*-*IV*	-	2.5	0.6	-	16 (9.8)	-	33 (20.2)	-
	70	100%	-	-	100%^d^		-	8.6	0	-	6 (8.7)	-	11 (15.9)	-
Ramautarsing 2011 [36]	20	45%	100%^c^	-	100%^a^	*Grade III*	-	5	5	-	-	-	-	-
Santini-Oliveira 2014 [34]	27	100%	-	-	100%^a^	*Grade I/II*	-	11.1/7.4	3.7/7.4	-	2 (3.8)	-	(14.3)	-
	26	100%	97.9%^a^	-	100%^d^	*Grade I/II*	-	15.4/7.7	7.7/3.9	-	2 (3.8)	-	(11.5)	-

n: ^a^subgroup of total study population, ^b^components of subgroup; (c)ART (combined) antiretroviral therapy; ANC antenatal care, PMTCT prevention of mother-to-child transmission; NRTI nucleoside reverse transcriptase inhibitor: ^a^lamivudine/zidovudine, ^b^ other, ^c^not specified, ^d^stavudine; NNRTI non nucleoside reverse transcriptase inhibitor: ^a^neviparine (NVP), ^b^other, ^c^not specified, ^d^single dose; PI protease inhibitor: ^a^LPV/r or other first generation drug PI ^b^other, ^c^not specified, ^d^LPV/r increased dose of >800/200 mg/day), ^e^second generation PI; Atazanavir; TC total cholesterol, TG triglycerides, HDL-C high density lipoprotein-cholesterol; Pregnancy outcome; PTB preterm birth, LBW low birth weight, PE preeclampsia; ^*^Duran et al. had data on ART in 293 cases, and data on adverse events (dyslipidemia) in 271 cases; Grading of dyslipidemia: Grade I-IV according to DAIDS [43]; UNS unspecified

Three studies reported a not further defined ‘incidence of dyslipidaemia’ of 0–7.5% [31, 32, 41]. Areechokchai et al. [41] found three cases (1.2%) of dyslipidaemia in participants on a first generation PI-based regimen (indinavir). The other two studies did not mention the type of ART used and reported no cases of dyslipidemia [31, 32].

The highest dyslipidaemia rates were reported by studies that recorded an incidence of dyslipidaemia of TC >200–240 mg/dl or TG >150–250 mg/dl (Table 2) [26, 28, 42]. Agostini et al. [28] found more dyslipidaemia among participants on a PI- than on a NNRTI- or nucleoside reverse transcriptase inhibitor (NRTI)-based ART regimen. Floridia et al. [26] observed an association between nevirapine use and higher HDL-C levels, between PI-based ART and higher TC, HDL-C, and TG levels, and between stavudine treatment and higher TG concentrations. Calza et al. [42] found no association between first generation PI LPV/r serum concentration and the incidence of hyperlipidaemia.

Other studies reported the incidence of dyslipidaemia expressed in severity scores of adverse events - Grade I, TC 200 to <240 mg/dl or TG 150–300 mg/dl; Grade II TC 240 to <300, TG >300 to 500; Grade III TC ≥ 300 or TG >500 to <1000, and Grade IV TG >1000 mg/dl – and mostly found cases of lower grades of dyslipidaemia [33, 34, 36, 37, 43, 44]. Duran et al. [37] found dyslipidaemia cases in all treatment groups and two cases (0.6%) hypertriglyceridemia grade II in patients receiving first generation PI nelfinavir. Studies that compared conventional doses of LPV/r to increased doses of LPV/r found higher rates of dyslipidaemia in the increased dose groups [33, 34, 44]. Ramautarsing et al. [36] found an incidence of 5% hypercholesterolemia and 5% hypertriglyceraemia in participants on first generation PI-based ART.

Two studies, [32, 42] had a control group consisting of non-pregnant HIV-infected women (*n* = 170). Calza et al. [42] found that the incidence of hypercholesterolemia was higher in the pregnant group than in the non-pregnant group (6 (29%) vs. 4 (20%)), while the incidence of hypertriglyceridemia was lower in the pregnant group compared to the non-pregnant group (*n* = 10, 48% vs. *n* = 11, 55%).

### Impact of duration since start ART on dyslipidaemia

Four studies reported the incidence of dyslipidaemia in relation to the duration of ART before conception. Floridia et al. [26] found an association between being treatment-naïve at conception and lower TG and higher TC concentrations during pregnancy. In accordance, Agostini et al. [28] found that 12 out of 17 (70.6%), and 14 out of 17 (82.4%) participants that were on ART at conception versus 6 out of 12 (50%) and 4 out of 12 (33.3%) participants that started PMTCT during pregnancy developed hypercholesterolemia and hypertriglyceridemia, respectively. Areechokchai et al. [41] only found cases of dyslipidaemia (3 (1.2%)) in participants that were on combined ART at conception.

### Pregnancy outcomes

No studies related serum lipid concentrations or the incidence of dyslipidaemia to pregnancy outcomes.

## Discussion

In this systematic review we observed a physiologic increase of serum lipid concentrations and widely variable rates of dyslipidaemia among HIV-infected pregnant women over the duration of pregnancy and across the included studies. The studies reported a higher rate of dyslipidaemia and serum lipid concentrations in women who were treated with first generation PI-based ART compared to women treated with an NRTI or NNRTI treatment regimen, and in women who were on ART at conception compared to women who only started ART during gestation. There was insufficient data and substantial heterogeneity which impair the ability to draw strong conclusions on the association of serum lipid concentrations in HIV-infected pregnant women and pregnancy outcomes, and compare serum lipid concentrations throughout pregnancy to concentrations in HIV-uninfected women.

In non-pregnant study populations TC, HDL-C and LDL-C decrease and TG increases directly following HIV-infection, but TC, LDL-C and TG increase after ART is started [16]. The extent of the change in serum lipid concentrations is most evident in the TG concentrations and under first generation PI-based and NNRTI-based ART [16]. Previous studies found increases in all lipids in the first 12 months on first generation PI-based ART. NNRTI-based ART was associated with milder increases [45, 46]. ART use has also been associated with dyslipidaemia in pregnancy; especially treatment with PIs caused considerable increases in serum lipid concentrations [47]. A previous study by Floridia et al. [24] found a significant increase in lipid levels in HIV-infected women that received PI-based ART during pregnancy. This finding was supported by studies included in this review that compared PI use to other ART regimens [26, 28, 30]. The highest serum lipid concentrations found in this review were derived from studies that used first generation PI-based ART regimens [27, 29, 30, 33, 37, 40, 42], while the lower values did not use PIs, or used second generation PIs [27, 30, 31].

HIV-infection did not seem to change the physiologic increase in serum lipids during pregnancy [44, 45]. The only study that compared lipids in all three trimesters found a larger increase in lipid concentrations in HIV-uninfected, compared to HIV-infected women [36]. Although the other reported serum lipid values were individual measurements, the overall increases in serum lipid concentrations over pregnancy fell within the ranges of a normal pregnancy [48, 49]. A meta-analysis by Spracklen et al. [15], showed that women who develop PE experienced higher serum lipid concentrations of TC and TG during all trimesters of pregnancy compared to normotensive women. Two other recent studies confirmed the association between higher serum TG concentrations and the development of HDPs [16, 17]. In this review one study, [28] found two cases of PE in HIV-infected patients on ART with hypertriglyceridemia. In theory, dyslipidaemia caused by HIV or ART in pregnancy could lead to hypertensive disorders of pregnancy (HDP). Whether HIV and ART are directly associated with HDP is still controversial due to the quality of the evidence [50].

A strength of our systematic review is the elaborate search strategy that was applied in five electronic databases, maximizing chances of finding all eligible publications and strict adherence to the PRISMA guidelines including risk of bias assessment to systematically categorize and review the collected data.

Nevertheless, a number of limitations need to be considered in interpreting these findings. First, the heterogeneity in outcome definition among the studies included in this review complicated drawing conclusions on our outcome measure. Since almost half of the studies did not specify their outcome measure beyond the grading of dyslipidaemia, the exact measure of the outcome we were interested in could not be assessed and a meta-analysis on serum lipid concentrations throughout pregnancy could not be performed. The lack of HIV-uninfected control groups in the studies included in this review limited our ability to assess the contribution of HIV-infection to changes in serum lipid concentration during pregnancy and the consequences for pregnancy outcome. The only two studies that compared an HIV-infected to an HIV–uninfected group found lipids to be higher in the uninfected group [35, 40].

Second, our review is limited by the suboptimal quality of the reported data. No studies mentioned controlling for missing data and only a few studies considered confounders such as age, BMI, dietary patterns, socio-demographics or CD4 count. BMI is an important potential confounder, as previous studies have found that BMI is a risk for the development of PE as part of the metabolic syndrome [51, 52]. Likewise, dietary patterns such as the Mediterranean diet are negatively associated with the metabolic syndrome [53]. Since 5 out of 17 studies originate from Italy, lower reported outcomes might have reflected the dietary pattern in the region. The lack of data on these possible confounding factors could have unpredictable ways (either underestimate or overestimate). Future studies are recommended to consider these confounders including monitoring of dietary patterns among participants.

Despite improvements in treatment of HIV-infection and prevention of PMTCT, pregnant women living with HIV should receive dedicated antenatal care. Not only do these women face the physiological changes in pregnancy, they also have a risk of developing complications through HIV-infection or ART, [47] resulting in an overall increased morbidity and mortality during pregnancy [54, 55]. Although the values of serum lipid concentrations fell within the reference range of serum lipid concentrations observed in non-HIV infected pregnancies, in this systematic review we found a higher rate of dyslipidaemia and serum lipid concentrations in the groups that were treated with first generation PI-based ART and in the groups that were on treatment at conception. The potential association between dyslipidemia and HDPs and PE suggests a clinical value to monitoring serum lipid concentrations in these subpopulations as part of optimal management. Currently, treatment options for dyslipidaemia in pregnancy are primarily limited to dietary and lifestyle changes, with statins not recommend in routine practice because of their potential teratogenicity [19]. Recently, omega-3 fatty acids have been found effective and safe in pregnancy to reduce TG levels [20]. Further research into the individual effects of HIV-therapeutics on dyslipidaemia in pregnancy could aid clinical practice in the management of (dyslipidaemia) risk factors and therapeutic decision making for HIV-infected pregnant women.

## Conclusion

This systematic review found physiologic concentrations of serum lipids for women living with HIV and for women receiving ART during pregnancy. Serum lipids were increased in users of first generation PI-based ART and for those on treatment at conception. Future studies are needed that include HIV-uninfected control groups and adequately control for potential confounders.

## Additional files

Additional file 1: Search strategy. (DOCX 78 kb)

Additional file 2: Quality assessment for individual studies. (DOCX 136 kb)

## Abbreviations

ANC: Antenatal care
ART: Antiretroviral therapy
GWG: Gestational weight gain
HDP: Hypertensive disorders of pregnancy
HIV: Human immunodeficiency virus
LBW: Low birth weight
LDL-C: Low-density lipoprotein cholesterol
MTCT: Mother-to-child transmission
NNRTI: Non-nucleoside reverse transcriptase inhibitors
NRTI: Nucleoside reverse transcriptase inhibitor
NVP: Neviparine
PE: Preeclampsia
PI: Protease inhibitor
PMTCT: Prevention of mother-to-child transmission
PTB: Preterm birth
SSA: Sub-Saharan Africa
TC: Total cholesterol
TG: Triglycerides
