# Supplementary material for: The association between HIV (treatment), pregnancy serum lipid concentrations and pregnancy outcomes: a systematic review
Source: BMC Infect Dis. 2017 Jul 11;17:489. doi: 10.1186/s12879-017-2581-8 (PMC5505132; doi:10.1186/s12879-017-2581-8)
Supplement: Supplementary file 1 — Search strategy. (DOCX 78 kb) [file 12879_2017_2581_MOESM1_ESM.docx]

| **Additional file 2. Search Strategy** | |  |
| --- | --- | --- |
| Search | Terms | Limit |
| #1 | Pregnan* OR expecting OR “pregnancy”[MeSH Terms] OR maternal OR obstetric OR intrapartum OR antepartum OR gestation | Title/Abstract |
| #2 | HIV OR HIV[MeSH Terms] OR HIV seropositivity[MeSH Terms] OR HIV infection OR HIV infections[MeSH Terms] OR AIDS OR acquired immuno deficiency syndrome[MeSH Terms] OR acquired immunodeficiency syndrome virus* OR acquired immune deficiency syndrome virus* OR AIDS virus* OR HTLV-III OR human immunodeficiency virus* OR human T cell lymphotropic virus type III OR human T-cell lymphotropic virus type III OR human T lymphotropic virus type III OR human T-lymphotropic virus type III OR human T cell leukemia virus type III OR human T-cell leukemia virus type III OR LAV-HTLV-III OR lymphadenopathy-associated virus OR lymphadenopathy associated virus | Title/Abstract |
| #3 | antiretroviral therapy, highly active[MeSH Terms] OR HAART OR highly active anti retroviral therap* OR highly active antiretroviral therap* OR antiviral agents[MeSH Terms] OR anti-HIV OR anti-AIDS OR anti-retroviral* OR anti retroviral* OR antiviral* OR protease inhibitor* OR PIs OR endopeptidase inhibitor* OR nucleoside reverse transcriptase inhibitor* OR NRTI* OR NNRTI* OR Protease antagonist* OR antiprotease OR peptidase inhibitor* OR protease inhibitor* OR reverse-transcriptase inhibitor* OR reverse transcriptase inhibitor* OR fusion inhibitor* OR entry inhibitor* OR integrase inhibitor* | Title/Abstract |
| #4 | abacavir OR didanosine OR dideoxyinosine OR emtricitabine OR lamivudine OR stavudine OR tenofovir OR zidovudine OR azidothymidine OR delavirdine OR efavirenz OR etravirine OR nevirapine OR rilpivirine OR atazanavir OR darunavir OR fosamprenavir OR indinavir OR nelfinavir OR ritonavir OR saquinavir OR tipranavir OR enfuvirtide OR maraviroc OR dolutegravir OR elvitegravir OR raltegravir OR cobicistat OR ABC OR FTC OR 3TC OR D4T OR TDF OR AZT OR ZDV OR DLV OR EFV OR ETR OR nvp OR rpv OR atv OR drv OR fos-apv OR FPV OR IDV OR NFV OR RTV OR SQV OR TPV OR T-20 OR MVC OR DTG OR EVG OR RAL OR COBI OR ZIagen OR videx OR emtriva OR epivir OR zerit OR viread OR retrovir OR rescriptor OR sustiva OR intelence OR viramune OR edurant OR reyatez OR prezista OR lexiva OR crixivan OR viracept OR norvir OR invirase OR aptivus OR fuzeon OR selzentry OR tivicay OR vitekta OR isentress OR tybost OR epzicom OR triumeq OR trizivir OR evotaz OR prezcobix OR atripla OR stribild OR complera OR truvada OR combivir OR kaletra | Title/Abstract |
| #5 | cholesterol* OR lipid* OR lipids[MeSH Terms] OR density lipoprotein* OR HDL OR LDL OR VLDL OR triglyceride* OR dyslipid* OR dyslipidemias[MeSH Terms] OR hyperlipid* OR hypertriglycerid* OR hypercholesterol* OR fatty acid* | Title/Abstract |
| Combination | **#1 AND #2 OR #3 OR #4 AND #5** |  |
