# Supplementary material for: The association between HIV (treatment), pregnancy serum lipid concentrations and pregnancy outcomes: a systematic review
Source: BMC Infect Dis. 2017 Jul 11;17:489. doi: 10.1186/s12879-017-2581-8 (PMC5505132; doi:10.1186/s12879-017-2581-8)
Supplement: Supplementary file 2 — Quality assessment for individual studies. (DOCX 136 kb) [file 12879_2017_2581_MOESM2_ESM.docx]

| **Additional file 3. Quality assessment for individual articles** | | | | | | | | | | | |
| --- | --- | --- | --- | --- | --- | --- | --- | --- | --- | --- | --- |
|  |  |  |  |  |  |  |  | **SImilarity study groups** | | |  |
| Study | Study design | Blinding | Selection of study population | Completeness of data | Origin of data (database or measurements) | Definition of outcome | Confounders taken into account? | Similarity of ART used | Lipids measured? | dyslipidemia as clinical outcome assessed? | Total points for quality assessment out of 16 |
| Agostini, 2008 | Case series | NA | Not described whether individuals selected to participate in the study are likely to be representative of the target population: Unclear Risk | No missing outcome data:  Low Risk | Sample of HIV+ pregnants from clinical files:  High risk | No definition of laboratory measurements, pre-eclampsia or pancreatitis High risk | No confounders were taken into account. High risk | Unclear Risk | N | Y | 6 |
| Areeckokchai, 2009 | Cohort study | NA | Only included women with asymptomatic HIV infection and no concomitant disease. High risk | No missing outcome data: Low Risk | Assessment occured through self-report  High Risk | Definitions stated  Low Risk | Multivariate analysis on confounders  Low Risk | Unclear Risk | N | N | 7 |
| Bonafe, 2013 | RCT: drug safety study | No, but unlikely to influence outcome Low Risk. | Participants selected representative of target population.  Low Risk | Participants excluded TG>500 mg/dL, or other grade III AEs  High Risk | Own measurements. Low Risk | Primary outcome and endpoints stated.  Low Risk | Confounders tested in logistic regression.  Low Risk | Low Risk | Y | UR | 13 |
| Cade, 2015 | Cross sectional observational study | NA | Participants selected representative of target population. HIV- women from women's health clinic  Low Risk | No missing outcome data:  Low Risk | Own measurements. Low Risk | Aims and definitions stated.  Low Risk | Confounders mentioned, but not accounted for in analysis.  High Risk | Low Risk | Y | N | 12 |
| Calza, 2012 | observational, open label study | NA | HIV+ and HIV- participants are drawn from same community (hospital) and are representative of target population.  Low Risk | No missing outcome data: Low Risk | Own measurements. Low Risk | Aim of the study stated and definition of trough plasma concentrations given. Low Risk | Multiple regression performed for possible confounders BMI and albumin concentrations. Low Risk | Low Risk | Y | Y | 16 |
| Duran, 2006 | retrospective cohort study | NA | All pregnancies within time frame of cohort included; representative of target population. Low Risk | Missing data might be of influence for outcome  High Risk | Medical charts reviewed. High Risk | Epidemiological features and toxicity related to ART not further defined. High Risk | Possible confounder; no data on AEs from mothers not receiving ARV, AIDS diagnosis not excluded. Retrospective data. High Risk | Low Risk | N | Y | 6 |
| El-Beitune, 2006 | prospective cohort study | NA | Sample representative of ARV-naive population. Exposed/non-exposed group are comparable.  Low Risk | No missing outcome data  Low Risk | Own measurements. Low Risk | The effect of ARV on triglycerides' not further defined. High Risk | Homogeneous patient sample to control for confounders. Did not take viral load, CD4 count into account. Unclear Risk | Low Risk | Y | N | 12 |
| Floridia, 2009 | prospective cohort study | NA | Sample representative of routine clinical care of HIV+ pregnant women in Italy. Low Risk | Missing outcome data on lipids might be related to outcome  High Risk | Data extracted from general database. High Risk | Study objective and definitions of hyperlipidemia given. Low Risk | Role of cofactors assessed with univariate and multivariable analyses Low Risk | High risk | Y | Y | 10 |
| Floridia, 2014 | prospective cohort study | NA | Sample representative of routine clinical care of HIV+ pregnant women in Italy. Low Risk | Missing outcome data on lipids might be related to outcome. High Risk | Data extracted from general database. High Risk | Definitions of outcomes of interest given. Low Risk | Multivariate analysis for covariates *P<*0,05 in univariate analysis. Low Risk | Low Risk | Y | N | 10 |
| Livingston, 2007 | observational cohort study | NA | Sample representative of routine clinical care of HIV+ pregnant women on stable ARV therapy in the US. Low Risk | Missing outcome data on lipids might be related to outcome (dyslipidemia). High Risk | Data extracted from general database. High Risk | Definitions of outcomes of interest given. Low Risk | Confounders not mentioned. CD4 count for example not corrected for in statistical analysis. High Risk | Low Risk | Y | N | 8 |
| Luzi, 2013 | prospective cohort study | NA | HIV+ and HIV- paricipants selected from routine care clinic; representative of population. Low RIsk | No missing outcome data: Low Risk | Own measurements. Low Risk | Definitions of outcomes of interest given. Low Risk | Regression diagnostics performed to test for effects of collinearity. Low Risk | Low Risk | Y | N | 14 |
| Machado, 2013 | cross-sectional study | NA | Patients from university's reference center for treatment of women infected with HIV during pregnancy; routine clinical care. Low Risk | Missing data or insufficient serum for analysis in 2-4 cases. High Risk | Own measurements. Low Risk | No clear definition on 'normal range' for lipids High Risk | No confounders mentioned or taken into account. High Risk | Unclear Risk | N | Y | 7 |
| Nasi, 2011 | prospective cohort study | NA | HIV+ women from an Italian prospective cohort study on efficacy and toxicity of ARV during pregnancy. Controls age-matched who had a CS. Representative of population? High Risk | Insufficient reporting of attrition/exclusions to permit judgement of ‘Low risk’ or ‘High risk’. Unclear Risk | Own measurements. Low Risk | No clear definition on outcomes 'drug toxicity quantification in mtDNA'. High Risk | Regression diagnostics performed to test/adjust for effects of collinearity. Low Risk | Low Risk | Y | N | 9 |
| Omo-Aghoja, 2010 | prospective cohort study | NA | Sample and controls representative of population; matched on socio-economic status. Low Risk | No missing outcome data: Low Risk | Own measurements. Low Risk | No definitions of haema-talogical and biochemical changes or disease severity. High Risk | Matched on socio-economic status. no other confounders mentioned. High Risk | Unclear Risk | Y | N | 8 |
| Peixoto,2011 | prospective cohort study | NA | Sample from multi-center Latin-American cohort study under routine clinical care.  Low Risk | Missing outcome data on several outcome variables.  High Risk | Data extracted from general database.  High Risk | No definitions of 'adverse events'.  High Risk | No confounders mentioned or taken into account.  High Risk | High risk | Y | Y | 6 |
| Ramautarsing, 2011 | interventional open label trial | NA | Representative of the target population? Not described. Unclear Risk | No missing outcome data:  Low Risk | Own measurements. Low Risk | Definitions of outcomes of interest given. Low Risk | Only ‘weight’ in sensitivity analysis. Others not taken into account. High Risk | Low Risk | N | Y | 11 |
| Santini-Oliveira, 2014 | interventional open label trial | NA | Representative of the target population? not described. Unclear Risk | non-response rate similar for both treatment arms. Low Risk | Own measurements. Low Risk | Definitions of outcomes of interest given. Low Risk | No confounders mentioned or taken into account.  High Risk | Low Risk | N | Y | 11 |
| Abbreviations: ART antiretroviral therapy; LR low risk, allocated 2 points; UR unclear risk, allocated 1 point; HR high risk, allocated 0 points; N no, allocated 0 points; Y yes, allocated 2 points; NA not applicable | | | | | | | | | | | |
